# Nutritional and Microbial Responses of *Pocillopora verrucosa* to Co‐Culture With *Chromis viridis* Damselfish

**DOI:** 10.1111/1758-2229.70291

**Published:** 2026-02-06

**Authors:** Rachel C. Neil, Jonathan A. Barton, Andrew Heyward, David S. Francis, Leo Nankervis, Thomas S. Mock, Craig Humphrey, David G. Bourne

**Affiliations:** ^1^ College of Science and Engineering James Cook University Douglas Queensland Australia; ^2^ Australian Institute of Marine Science Cape Cleveland Queensland Australia; ^3^ AIMS@JCU, James Cook University Townsville Queensland Australia; ^4^ The National Sea Simulator, Australian Institute of Marine Science Cape Cleveland Queensland Australia; ^5^ Australian Institute of Marine Science, Indian Ocean Marine Research Centre, University of Western Australia Crawley Western Australia Australia; ^6^ Nutrition and Seafood Laboratory (NuSea.Lab), School of Life and Environmental Sciences Deakin University Geelong Victoria Australia; ^7^ Centre for Sustainable Tropical Fisheries and Aquaculture James Cook University Townsville Queensland Australia

## Abstract

Associations with fish can benefit corals by increasing growth and stress tolerance. To investigate microbial and nutritional responses of corals to fish associations in the context of enhancing coral aquaculture outcomes, *Pocillopora verrucosa* were cultured for 3 months with different combinations of live feeds and schools of juvenile *Chromis viridis* damselfish. The combined live feeds and fish treatment resulted in a bacterial community most similar to wild *P. verrucosa,* dominated by *Endozoicomonas‐*affiliated taxa. Protein content was enhanced in corals with access to live feeds and/or dissolved fish wastes compared to unfed controls. Total lipid concentrations were elevated in captive corals with access to dissolved fish wastes and at moderate levels in those supplied live feeds, likely due to the activity of corals' symbionts and deposition of derived lipids from live feeds, respectively. However, all captive corals demonstrated a significant reduction in storage lipid concentration compared to samples from the wild. Fatty acid analysis indicated these shifts were likely the result of higher light levels in the field supporting Symbiodiniaceae photosynthesis and potentially feeding on wild zooplankton. Co‐culturing captive corals with fish and providing appropriate live feeds may therefore offer an effective approach to improve coral nutrition, health and microbiome stability.

## Graphical abstract

Assessing the microbiome of captive *Pocillopora verrucosa* corals showed that populations of proposed microbial symbiont *Endozoicomonas* could only be maintained when corals were co‐cultured with *Chromis viridis* damselfish and supplied live feeds. Therefore, utilising fish co‐culture in coral aquaculture may be useful to support coral microbial populations.

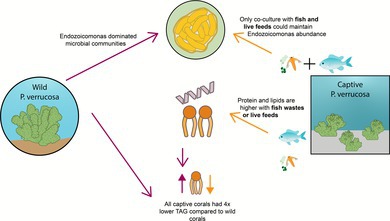

## 1 Introduction

Sustainable coral production in aquaculture systems is essential to support reef restoration efforts and meet the growing demand from the ornamental industry (Barton et al. 2017; Randall et al. 2020). Enhancing the survival and growth of asexually produced coral propagules is critical to achieving production targets. Key environmental conditions such as light, water quality, fouling control, and nutrient supply (both heterotrophic and inorganic) are all vital factors for successful coral cultures (Ferrier‐Pagès et al. 2003; Forsman et al. 2011; Houlbrèque et al. 2003; Neil et al. 2021). Although some corals can derive up to 90% of their energy requirements from translocated photosynthates and direct feeding on their algal endosymbiont partner Symbiodiniaceae, heterotrophic feeding often enhances coral growth and survival (Conlan et al. 2017; Falkowski et al. 1984; Ferrier‐Pagès et al. 2003; Wiedenmann et al. 2023). More robust and consistent production may also be facilitated by exogenous nutrition, as heterotrophic feeding can increase coral resistance to environmental stressors or support important bacterial communities within the holobiont (Ferrier‐Pagès et al. 2010, 2003; Galand et al. 2020; Toh et al. 2014).

Studies on coral nutrition highlight the importance of tailoring the composition and delivery of nutrients to suit individual species, therefore maximising uptake while minimising cost (Conlan et al. 2019; Ding et al. 2021; Osinga et al. 2011; Tagliafico et al. 2018). Most captive systems rely on easily mass‐produced feeds such as enriched *Artemia*, rotifers, microalgae or commercial diets (Borell et al. 2008; Petersen et al. 2008; Séré et al. 2010; Toh et al. 2014), though diets with a greater diversity of live feeds have been shown to have benefits for coral health and growth (Conlan, Bay, et al. 2018; Conlan et al. 2017). Similarly, aspects of the physical environment, including dissolved nutrients and the light available for photosynthesis, will impact production and subsequent translocation of photosynthates from Symbiodiniaceae symbionts to coral hosts (Morris et al. 2019; Treignier et al. 2008). Nutrient delivery in coral aquaculture is therefore a complex balance of heterotrophic inputs and physical conditions for symbiont activities, which can be challenging to disentangle.

Alongside nutritional strategies, culture practices are also an important consideration in the implementation of coral aquaculture ventures. Integrated multi‐trophic systems are gaining prominence in the wider aquaculture industry, which involve growing multiple species in linked or mixed mesocosms rather than in monoculture (Barrington et al. 2008; Knowler et al. 2020; Ridler et al. 2007). In these systems the specific biological properties or trophic niches of complementary species can be harnessed for improved production and nutrient capture, which has a positive impact on the economic and environmental feasibility of culture systems (Hala et al. 2024). On reefs, close association with fish has been documented to improve coral growth (Holbrook et al. 2008; Liberman et al. 1995; Meyer and Schultz 1985), photosynthetic rates (Garcia‐Herrera et al. 2017), and tolerance to thermal stress (Chase et al. 2018; Shantz et al. 2022) or sedimentation (Chase et al. 2020). These benefits have been attributed to two main mechanisms: Increased water movement resulting from the fishes' swimming and enhanced nutrient transfer from fish wastes (recently reviewed by Carmignani et al. (2025)). Fish swimming activity enhances water flow through coral colonies, reducing the diffusive boundary layer and in turn increasing oxygen and nutrient availability, improving photosynthesis (Garcia‐Herrera et al. 2017; Goldshmid et al. 2004; Liberman et al. 1995). Fish also excrete dissolved nitrogen and phosphorous, which are limiting nutrients for coral endosymbiont growth and photosynthesis, in forms that are readily assimilated by corals and their symbionts (Carmignani et al. 2025). Some studies have also suggested that corals may actively feed upon the particulate organic matter (POM) provided by fish faeces (Meyer et al. 1983; Shantz et al. 2022). As such, integrating fish into coral aquaculture could present an approach to improve physiological performance and nutritional condition of corals, thereby supporting coral health and increasing production.

When assessing the effects of different diets or culture conditions on corals, it is important to consider multiple aspects of the coral holobiont response. Whilst survival and growth are the primary metrics producers look to optimise (outside of colouration for the ornamental trade), factors such as microbiome composition and nutritional status, including energy stores, should be considered given their direct influence on coral performance. The two most energy dense macronutrients, lipid and protein, represent important energy sources for coral growth and reproduction, and are utilised to help the animal survive during periods of stress such as bleaching (Grottoli and Rodrigues 2011; Houlbreque and Ferrier‐Pages 2009). The coral microbiome also plays an important role in nutrient cycling and stress resilience (Krediet et al. 2013; Voolstra et al. 2024). Changes to coral microbial communities can be indicative of stress, though they can also occur in response to external factors such as environmental conditions, with associated physiological implications for the coral host (Bourne et al. 2016; Voolstra et al. 2024). In turn the makeup of the microbiome will be influenced by the available heterotrophic diet (Galand et al. 2020). The interplay between host nutritional status and the associated microbiome is critical for the fitness of corals destined for restoration out‐planting or commercial export, both of which represent physiologically challenging events (Boch et al. 2019; Delbeek 2008; Gantt et al. 2023; Lirman 2000).

Previously we demonstrated that fish‐associated branching coral *Pocillopora verrucosa* displayed increased growth and symbiont density when supplied with live feeds or dissolved fish wastes from juvenile *Chromis viridis*, compared to unfed control corals (Neil et al. 2025). To better understand the underlying drivers of these changes and derive potential metrics for coral fitness for long‐term holding, out‐planting or commercial export, *Pocillopora verrucosa* fragments were further assessed for proximate, lipid class and fatty acid composition, in addition to microbial community composition. Importantly, the condition of cultured corals was compared to their in situ field state to evaluate physiological and microbial changes following acclimation to captive environments.

## 2 Methodology

### 2.1 Experimental Design

*Pocillopora verrucosa* nubbins were collected and cultured in experimental conditions as per Neil et al. (2025). Briefly, *P. verrucosa* were collected from Davies Reef (−18.825622, 147.626881) on the Great Barrier Reef and transported to the Australian Institute of Marine Science's National Sea Simulator (SeaSim). Colonies were fragmented into ~10 g nubbins, which were allowed to recover and acclimate for 1.5 months to captive conditions. Following acclimation, coral nubbins were assigned to one of six treatments, across twenty‐four 50 L experimental tanks (four replicates per treatment), and maintained for 3 months in an indoor, controlled environment. Experimental treatments were: ‘LiveFeeds’ supplied with mixed live feeds, ‘Fish’ co‐cultured with 10 juvenile *Chromis viridis* damselfish being fed a pellet diet, ‘Dissolved’ supplied with water from a tank of 10 juvenile *C. viridis* passed through a 50 μm filter, ‘LiveFeeds + Fish’ co‐cultured with 10 juvenile *C. viridis* while also given a supply of mixed live feeds, ‘Pellets’ supplied only with the pellet diet fed to the fish, and ‘Control’ kept with no fish and supplied no feeds. The mixed live feeds consisted of polyunsaturated fatty acid (PUFA) enriched *Artemia* nauplii (0.5 nauplii mL^−1^), rotifers (0.5 rotifers mL^−1^) and a mix of microalgae (*Nannochloropsis oceania, Tisochrysis lutea, Chaetoceros muelleri, Dunaliella* sp., *Proteomonas sulcata*; 2000 algae cells mL^−1^). Coral feeds were given once a day, whilst fish schools were fed twice daily with 0.12 g of Aquaforest AF Tiny Fish Feed.

Tanks received 0.1 μm filtered seawater at 28°C ± 0.1°C, at a rate of 0.8 L min^−1^. Water movement within the tanks was provided by Turbelle nanostream 6015 circulation pumps (Tunze Aquarientechnik, Penzberg, Germany). Light was supplied by one LED light (Hydra 52, AquaIllumination, Bethlehem, USA) for each tank from 0830 to 1630 at 150 μ mol cm^−2^ s^−1^, with one‐hour ramps at sunset and sunrise.

### 2.2 Sample Collection

*P. verrucosa* fragments were sampled at 3 timepoints for biochemical and microbial analysis: (i) Upon arrival at the SeaSim after collection from the wild (‘Field’), (ii) after fragmentation and the 1.5 months acclimation period, just prior to the start of the experiment (‘Post‐acclimation’), and (iii) after 3 months subjected to the experimental treatments (‘Treatment’).

For microbial analyses, branch tips (which included skeleton, tissue and mucus) were collected and stored in salt‐saturated pH 8.0 DMSO‐EDTA at −20°C. Water samples (50 mL) for microbial analysis were passed through a cell strainer then filtered through a 0.22 μm filter, and frozen at −80°C. Water was sampled in triplicate for Field and Post‐acclimation stages, and once per tank at the final timepoint (*n* = 4 per treatment).

For biochemical analysis, coral nubbins were patted with paper towel to remove excess water, wrapped in aluminium foil, secured in an airtight bag before being stored at −20°C in preparation for analysis of proximate, lipid class and fatty acid composition. *Artemia*, rotifer and microalgae samples were also collected by centrifuging aliquots from larger feed batches to concentrate the plankton, then removing excess water and freezing at −20°C.

### 2.3 Microbiome Analyses

DNA was extracted from coral tissues using a QIAGEN Blood and Tissue kit, with the Proteinase K digestion replaced with a bead beating step, using 4 × 1 mm stainless steel beads and 0.25 mL 0.7 and 0.15 mm garnet mix beat at 5.5 m s^−1^ for 30 s on a FastPrep‐24 5G. Cells from water samples (on 0.22 μm membranes) were first lysed using lysozyme, then the lysate removed from the filters by the addition of a lysis buffer and pressure from the syringe piston. Following this, DNA was extracted using the QIAGEN Blood and Tissue protocol supplied by the manufacturer. All extracted DNA samples were then purified using a Promega Wizard DNA Clean‐Up System. Samples were sequenced by the Ramacoitti Centre for Genomics (University of New South Wales, Sydney, Australia), using Illumina MiSeq 2x300 bp paired‐end sequencing targeting the 16S rRNA V3‐V4 region (341–805).

### 2.4 Biochemical Analyses

Coral and feed samples were processed and analysed as per Conlan et al. (2014). In brief, frozen samples were freeze‐dried (48 h) then crushed in toto using a 70 kN stainless steel hydraulic press. Ash content was measured by incineration in a muffle furnace for 18 h at 450°C. Protein content was determined via the Kjeldahl method (AOAC 2000) using an automated Kjeltech 8400 and a conversion factor of 6.25 (FOSS, Analytical Co. Ltd., Sweden). Total lipids were cold extracted from 2 g of homogenised sample using dichloromethane: methanol (2:1) for total lipid quantification (Folch et al. 1957). Lipid classes were analysed using an Iatroscan MK 5 s thin layer chromatography‐flame ionisation detector. Resulting chromatograms were then manually integrated using eDAQ PowerChrom v2.7.9 software to identify and quantify individual lipid classes relative to external standards (Sigma‐Aldrich Inc., St. Louis, USA and NuChek Prep Inc., Elysian, USA).

For fatty acid profiling, an aliquot of the extracted lipid sample underwent acid‐catalysed methylation to esterify fatty acids into methyl esters (FAME), with 100 μL of 23:00 (0.75 mg mL^−1^) added as an internal standard. FAME were analysed with a gas chromatography—flame ionisation detector (GC‐FID) (Agilent Technologies 7890A, USA) equipped with a J&W DB capillary column (60 m, 0.25 mm internal diameter, 0.15 mm film thickness); (Agilent Technologies, USA), a flame ionisation detector (FID), an Agilent Technologies 7693 autosampler injector, and a split injection system with helium as the carrier gas at 1.5 mL/min following the method presented in Francis and Turchini (2017). Individual fatty acid peak areas were corrected by theoretical relative FID response factors (Ackman 2002), and identified and quantified relative to external standards (Sigma‐Aldrich Inc., St. Louis, USA and NuChek Prep Inc., Elysian, USA) using GC ChemStation software (Agilent Technologies, USA).

### 2.5 Data Analysis

Bioinformatic processing of microbial data was performed via QIIME2 version 2023.9 (Bolyen et al. 2019) using the DADA2 pipeline (Callahan et al. 2016). Forward sequences were truncated to 255 bp and reverse to 240, and the first 11 and 6 bp were trimmed from forward and reverse sequences respectively. Representative ASVs were classified via a Naïve Bayes classifier trained using the V3–V4 region from the SILVA database (version 138.1, 99).

All further data analysis was conducted using R v4.3.1 (R Core Team 2023) and RStudio v2023.06.1 + 524 (Posit team 2023), where phyloseq was used to import and filter data (phyloseq (McMurdie and Holmes 2013)). Eukaryote, mitochondria and chloroplast sequences were removed from the feature table, then samples with less than 1000 reads were removed and amplicon sequence variants (ASVs) filtered to keep only taxa with ≥ 0.001% relative abundance in at least one sample. Samples were rarefied to 1026 reads, then observed richness and Shannon evenness were calculated (Lahti and Shetty 2012–2019) and compared using a Kruskal‐Wallis rank sum test followed by pairwise comparisons of estimated marginal means (emmeans (Lenth 2020)) from generalised linear mixed effects models (glmmTMB (Brooks et al. 2017)). PERMANOVA (vegan (Oksanen et al. 2024)) and indicator species analysis (indicspecies (De Caceres and Legendre 2009)) were used to compare community composition of corals within the different treatments, and ordination plots using redundancy analysis with ASV data standardised using the Hellinger method were used to visualise similarity in community structure.

Bayesian generalised linear mixed effects models were used to model proximate composition, lipid class and fatty acid class content of *P. verrucosa* fragments. Bayesian models were run via the brms package (Bürkner 2021) with Stan (Stan Development Team 2023). Although the link function and formula of models varied, in general treatment was treated as a fixed effect, with genotype, replicate tank and/or sample treated as blocking factors (see Table S1 for details). All model samplers were checked for well‐mixed traceplots, autocorrelation, chain convergence (R‐hat < 1.01) and effective sample size. Model fit was assessed using posterior probability checks and DHARMa residuals. Median and 95% credibility intervals (CI: calculated as 95% highest posterior density intervals) were then calculated, and Bayesian probability that the response of one treatment was greater than another was also calculated. For fatty acid data, analysis of similarity using Bray‐Curtis distances, pairwise PERMANOVA and indicator species analysis was used to identify differences between treatments, and principal components analysis was used to create ordination plots (factoextra (Kassambara and Mundt 2020)).

## 3 Results

### 3.1 Microbial Communities

**FIGURE 1:**
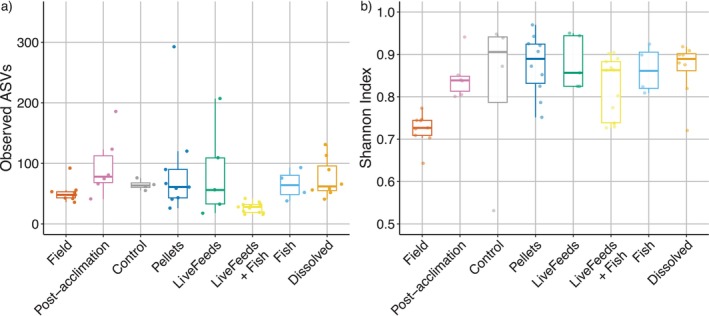
Boxplots of (a) species richness as observed ASVs and (b) evenness measured using Shannon Index.

A total of 9,044,994 high‐quality 16S rRNA amplicon reads were recovered from 140 samples, and following trimming and quality filtering 27, 598 ASVs were identified from 87 samples. Assessing diversity metrics across all treatments, there was significant differences in richness (Kruskal‐Wallis H (7) = 28.636, *p* value = < 0.001; Figure 1a) and evenness (Kruskal‐Wallis H (7) = 20.706, *p* value = 0.0042; Figure 1b) in the coral associated coral microbial communities. Field samples had a significantly lower Shannon Index than all the other coral samples within the captive environment treatments, indicating a less even distribution of species in these Field samples (Figure 1b; *p* values < 0.05). Post hoc tests revealed richness (i.e., observed number of ASVs) was significantly lower for corals from the LiveFeeds + Fish treatment compared to all other samples except the Control (Figure 1a; *p* values < 0.05).

**FIGURE 2:**
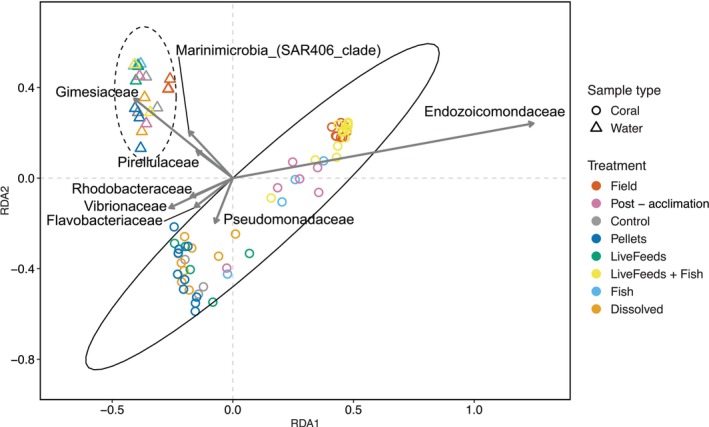
Redundancy analysis (RDA) ordination plot of coral and water bacterial community, with the key driving bacterial Family ASVs as arrows and 95% confidence intervals drawn as ellipses.

Coral associated bacterial communities were different from those found in their surrounding water (PERMANOVA with 10,000 permutations: *F* = 15.9934, *p* value < 0.001; Figure 2), and distinct bacterial communities were present between the corals from different treatments, Field and Post‐acclimation samples (PERMANOVA with 10,000 permutations: *F* = 4.3865, *p* value < 0.001). Dispersion also varied between the coral samples from the different treatments (PERMDISP with 10,000 permutations: *F* = 24.251, *p* value < 0.001). Field, Post‐acclimation, Fish and LiveFeeds + Fish corals showed grouping in the redundancy analysis, driven by *Endozoicomonas* abundance, and within this group the Field and Fish + LiveFeeds samples were tightly clustered (Figure 2). The remaining corals from the treatments Control, Pellets, Dissolved and LiveFeeds displayed a second grouping, driven by *Vibrionaceae, Rhodobacteraceae, Flavobacteriaceae* and *Pseudomonadacea* (Figure 2).

**FIGURE 3:**
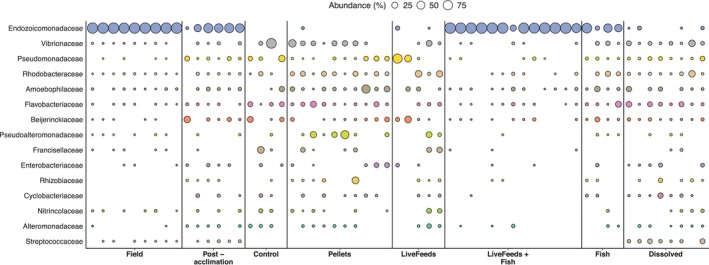
Sample by sample breakdown of the top 15 bacterial Families in microbial communities, as bubble plots of relative abundance. Note, sample replication is unequal between treatments due to some samples not amplifying or passing QA/QC or filtering steps.

Field coral communities were dominated by ASVs identified as belonging to the Family *Endozoicomonadaceae* (96.0% ± 2.5%), whilst all other identified bacterial families remained at ≤ 0.7% mean relative abundance. The Post‐acclimation corals showed a shift in microbial community composition, with the relative abundance of *Endozoicomonadaceae* affiliated reads lower at 51.9% ± 20.8% (Figure 3). Higher ASV richness was observed in these Post‐acclimation corals (Figure 1a), with a comparative increase in the relative abundance of ASVs affiliated with the families *Beijerinckiaceae* (7.3% ± 11.8%) and *Psuedomonadaceae* (4.4% ± 5.5%), and a general increase in mean relative abundance of other bacterial families, including *Amoebophilaceae, Peptostreptococcales‐Tissierellales* and *Cyclobacteriaceae*, to ~1%–2.7%.

After 3 months in the captive aquarium environment, there were no retrieved sequences affiliated with *Endozoicomonadaceae* in the Control corals (Figure 3). Additionally, corals from the LiveFeeds, Pellets and Dissolved treatments displayed low mean relative abundance of *Endozoicomonadaceae* affiliated reads (0.2%–1.9%). Only corals from the LiveFeeds + Fish and Fish treatments demonstrated *Endozoicomonadaceae* dominated microbial communities, with respective means of 89.5% ± 20.7% and 48.0% ± 29.8% of identified ASVs being associated with this family (Figure 3). This *Endozoicomonadaceae* dominance was relatively consistent across all samples from the LiveFeeds + Fish, though the relative concentration of these ASVs varied between individual Fish treatment corals (Figure 3).

Control coral samples demonstrated a higher relative abundance of *Vibrios* (22.4% ± 40.2%) than other treatments, though this was driven by one sample (Figure 3). Across all other treatments, *Vibrios* were present, ranging from a mean relative abundance of 0.2% to 11.6%, and were at their lowest in LiveFeeds + Fish. LiveFeeds had a higher mean abundance of *Rhodobacteraceae* affiliated reads than other corals, identified by indicator species analysis (12.5% ± 15.6%; *p* value = 0.0450), whilst *Pseudoalteromonadaceae* were at higher abundance in both Pellets and LiveFeeds samples (10.7% ± 17.2% and 5.66% ± 9.69%; *p* = 0.0472). Dissolved corals were characterised by a higher abundance of reads affiliated with *Streptococcaceae* (5.83% ± 5.13%; *p* value = 0.0007).

### 3.2 Proximate, Lipid and Fatty Acid Composition

**FIGURE 4:**
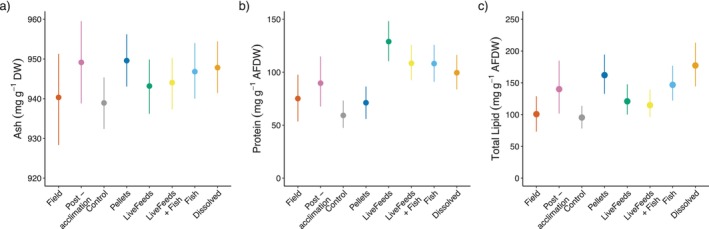
Proximate composition of sampled *Pocillopora verrucosa* , including (a) dry‐weight ash content, (b) ash‐free dry weight protein content, (c) ash‐free dry weight total lipid content. Points and bars represent modelled median and 95% credibility intervals.

The proximate composition of the sampled *Pocillopora verrucosa* varied across the Field, Post‐acclimation, and the experimental corals. Ash was slightly higher in the treatments where fish were present (Fish, Dissolved and LiveFeeds + Fish) and Pellets compared to Control (*p* > 86%; Figure 4a), though overall the absolute differences between coral ash content were small (< 10 mg g^−1^ DW (dry weight)).

Protein content was relatively low in Field corals (75.2 mg g^−1^ AFDW (ash free dry weight)), and though Post‐acclimation samples showed a higher mean concentration (89.7 mg g^−1^ AFDW) there was no evidence this represented a significant increase (*p* = 81%). Protein concentrations of treatment corals were highest in LiveFeeds (128.9 mg g^−1^ AFDW; *p* > 94%), but also elevated in Dissolved, Fish and LiveFeeds + Fish (~100–108 mg g^−1^ AFDW) compared to corals grown in the Control or Pellets treatments (~59–71 mg g^−1^ AFDW) (*p* > 99%; Figure 4b).

Total lipid concentration was lower in Field corals (100.7 mg g^−1^ AFDW) compared to Post‐acclimation (140.0 mg g^−1^ AFDW) (*p* = 94.5%). Lipid concentration was highest in Dissolved and Pellets (~162–177 mg g^−1^ AFDW), and both treatments where corals had access to live feeds had similar total lipid content (LiveFeeds and LiveFeeds + Fish; 120.9 and 114.8 mg g^−1^ AFDW respectively). Control corals had the lowest total lipid content (95.4 mg g^−1^ AFDW) out of all the treatment samples (*p* > 91%; Figure 4c).

**FIGURE 5:**
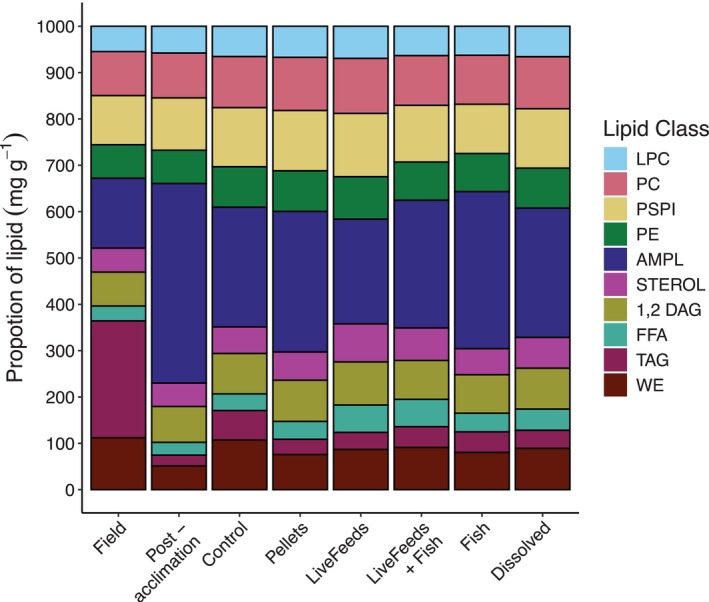
Relative proportions of different lipid classes within the total lipid fraction of *Pocillopora verrucosa*. AMPL, acetone mobile polar lipids; DAG, 1,2‐diacylglycerol; FFA, free fatty acids; LCP, lysophosphatidylchloline; PC, phosphatidylcholine; PE, phosphatidylethanolamine; PSPI, phosphatidylserine and phosphatidylinositol; Sterol, sterol; TAG, triacylglyceride; WE, wax esters.

Field corals were characterised by significantly higher storage lipid content (sum of WE (wax esters), TAG (triacylglycerols), FFA (free fatty acids) and 1, 2 DAG (diacylglycerols); 394.3 mg g^−1^ lipid) in comparison to any of the captive corals, driven primarily by a much higher concentration of TAG (252.0 mg g^−1^ lipid; Figure 5). Even when accounting for lower total lipid in Field samples compared to some treatments, TAG was significantly higher in Field samples than all other corals, with a modelled median of 24.7 mg g^−1^ AFDW of TAG compared to ~3–6 mg g^−1^ AFDW in the Post‐acclimation and treatment corals (*p* = 100%). Conversely, Post‐acclimation corals had the lowest storage lipid content (180.3 mg g^−1^ lipid) compared to all other samples (*p* = 100%), whilst Control corals had a higher proportion of storage lipids compared to other the treatments (*p* > 85%; Figure 5) except LiveFeeds + Fish (*p* = 77.2%). Due to lower storage lipids, corals from the LiveFeeds, Dissolved, Fish and Pellets treatments had a higher proportion of lipids that perform structural roles (Sterol, AMPL (acetone mobile polar lipid), PE (phosphatidylethanolamine), PSPI (phosphatidylserine/phosphatidylinositol), PC (phosphatidylcholine) and LPC (lysophosphatidylcholine)) lipids compared to Control corals (*p* > 85%).

**FIGURE 6:**
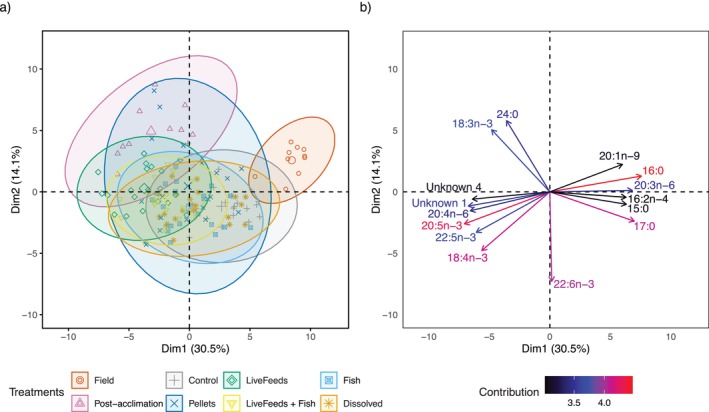
Principal components analysis of fatty acid classes from *Pocillopora verrucosa* , showing (a) ellipses of 95% confidence intervals for the different treatments and (b) the 15 most influential individual fatty acids and their contribution to the overall variance.

Fatty acid (FA) composition of corals significantly varied between the Field, Post‐acclimation and treatment groups (Figure 6a; PERMANOVA with 10,000 permutations: *F* = 20.112, *p* value < 0.001). However, pairwise PERMANOVA found that corals from the Dissolved, Fish and Pellets treatments had similar compositions (*p* values > 0.05), as did the samples from the Fish and LiveFeeds + Fish (*F* = 2.804, *p* value = 0.057). Saturated fatty acids (SFA), as a percentage of total fatty acids, were higher in Field corals compared to all other corals (*p* > 99%). Monounsaturated fatty acids (MUFA) were higher in both the Field and Post‐acclimation samples compared to treatment corals (*p* > 99%), whilst polyunsaturated fatty acids (PUFA; including all individual fatty acids with two or more double bonds between carbon atoms in the fatty acid molecule) were elevated in all captive corals compared to Field (*p* = 100%). Field and Post‐acclimation corals both had a lower n‐3 PUFA:n‐6 PUFA ratios (1.19 and 1.32 respectively) than any of the treatment corals (1.55–1.80, *p* > 96%). Field corals also had a slightly lower n‐3 LC PUFA:n‐6 LC PUFA ratio (1.48) compared to all treatment corals (~1.65–1.80; *p* > 89%) except Pellets, though Post‐acclimation corals had the lowest n‐3 LC PUFA: n‐6 LC PUFA of all at 1.22 (*p* > 97%). The LiveFeeds treatment had significantly lower SFA levels compared to all other treatment corals (*p* > 89%). LiveFeeds and LiveFeeds + Fish nubbins had higher PUFA concentrations compared to all other captive corals (*p* > 92%), though the Dissolved, Fish and Pellets treatments all had higher PUFA than Control (*p* > 98%).

Indicator species analysis showed Field corals' dissimilarity to captive corals was driven by higher concentrations of 20:1n‐9, 18:1n‐9, 16:0, 20:3n‐6, 20:0 and 18:1n‐9t, whilst Post‐acclimation had increased concentrations of 22:1n‐11, 24:0, 10:0, 18:0. All captive corals were associated with increased concentrations of 18:4n‐3, 20:5n‐3 (EPA), 20:4n‐6 (ARA) and 16:3n‐4, and treatment corals were also associated with 22:6n‐3 (DHA). LiveFeeds corals were associated with increased levels of 20:3n‐3 (Figure 6b), though 18:3n‐3 was associated with and at higher concentration in both the LiveFeeds and Post‐acclimation samples. Higher levels of 18:1n‐7 and 15:1n‐5 were associated with all corals with access to live feeds, namely, LiveFeeds, LiveFeeds + Fish and Post‐acclimation.

## 4 Discussion

Coral‐associated microbial communities underwent substantial shifts following translocation from the field to the aquarium environment. The microbiomes associated with Field *Pocillopora verrucosa* were dominated by *Endozoicomonadaceae* affiliated taxa, whilst Post‐acclimation fragments saw a reduction in the relative abundance of *Endozoicomonadaceae* and higher relative abundances of other bacterial families such as *Beijerinckiaceae* and *Psuedomonadaceae*. These shifts are likely attributable to the physiological challenges of fragmentation and transition to the new conditions in captivity. For example, the microbiome of *Siderastrea sidereal* were shown to shift then stabilise into a new community composition after 28 days in captivity (Pratte et al. 2015). The microbiome of the deep‐sea coral *Eguchipsammia fistula* changed significantly after 1 year in captivity, attributed to the differences in environmental conditions in captivity compared to the in situ samples (Röthig et al. 2017). *P. verrucosa* 's microbiome has been previously shown to be relatively stable when subjected to environmental stress (Hochart et al. 2023; Pogoreutz et al. 2018; Strudwick et al. 2022; Ziegler et al. 2019); however, fragmentation and transfer to the new environmental conditions encountered in an aquarium setting appear sufficient to overcome the typical robustness of *P. verrucosa* 's microbiome (Puntin et al. 2024; Strudwick et al. 2022).

Few sequences affiliated with *Endozoicomonadaceae* were recovered from coral fragments subjected to the Control, Pellets, LiveFeeds and Dissolved treatments. In addition the microbiome of corals in these treatments displayed elevated species richness, a response indictive of stress or opportunistic colonisation of pathogenic or non‐mutualistic bacteria (McDevitt‐Irwin et al. 2017; Meyer et al. 2014; Neave et al. 2016; Voolstra et al. 2024). This was supported by higher relative abundance of *Vibrionoaceae, Rhodobacteraceae, Pseudoalteromonadaceae and Streptococcaceae* affiliated taxa. Previous studies have reported some strains of *Vibrio* bacteria as pathogenic, which may opportunistically colonise corals and/or increase in abundance when corals are stressed (Tout et al. 2015; Vidal‐Dupiol et al. 2011). *Rhodobacteraceae* also commonly increase in compromised corals (Mouchka et al. 2010; Pollock et al. 2017). The corals of this study were in captive environments for 4.5 months, inclusive of the 3 months exposed to their respective treatments, thus the observed microbial community differences were most likely a response to the change in environmental conditions in which the corals were kept (Zheng et al. 2026). This indicates these captive conditions influenced the coral holobiont homeostasis and potential health status when compared to the coral's natural environment (e.g., different available nutrient profiles, light, water movement etc.) (Rosset et al. 2017).

Interestingly, the captive corals cultured with fish displayed higher relative abundance of *Endozoicomonadaceae* compared to those without. *Endozoicomonadaceae* in the Fish treatment had a similar relative abundance to the Post‐acclimation fragments, whereas the LiveFeeds + Fish samples were dominated by *Endozoicomonadaceae* and were the only captive corals to have similar observed abundance of this taxa compared to the field corals. *Endozoicomonas* bacteria have been widely reported as dominant in coral microbiomes, and in *Pocillopora* species they can make up > 75% of a coral's microbial community (Pogoreutz and Ziegler 2024). They are suggested to be part of a healthy microbiome, though questions remain around their exact function and the nature of their relationship with corals (Damjanovic et al. 2020; Hochart et al. 2023; Neave et al. 2016; Pogoreutz and Ziegler 2024). They form coral‐associated microbial aggregates (CAMAs), predominantly located in the gastrodermis in *P. verrucosa* (Neave, Rachmawati, et al. 2017), and are proposed to assist their host through nutrient cycling, amino acid synthesis and carbohydrate and protein provisioning (Maire et al. 2024; Neave, Michell, et al. 2017; Pogoreutz et al. 2022). The exact drivers of coral‐*Endozoicomonas* associations remain unclear, though our experiment demonstrated that direct fish‐coral association appears to play a role, as only the treatments with fish present in the tank with the corals retained high *Endozoicomonas* abundance (Pogoreutz et al. 2022; Pogoreutz and Ziegler 2024).

Whilst there have been no published studies looking at the effects of coral‐dwelling damselfish on their host coral's microbiome, past studies have explored similar relationships between clownfish and their anemone hosts (Pratte et al. 2018; Roux et al. 2019). These studies found that the skin/mucus microbiome of both is influenced by the presence of their partner, and if fishes separate from their host anemones their microbiome will shift to match that of non‐hosting fish (Pratte et al. 2018; Roux et al. 2019). However, these studies have not been able to determine the mechanisms behind these microbiome changes, outside of the obvious transmission of bacteria from the fish and anemone coming into contact with each other. Whilst in this experiment the *C. viridis* needed to be housed within the same tank as the *P. verrucosa* to maintain *Endozoicomonas* dominance, this affect is unlikely to be from direct contact, as *C. viridis* does not come into contact with their coral hosts as frequently as anemone fish (Parris et al. 2016). In addition, within native reef environments, adult *Chromis* biomass varies greatly between corals hosts, thus is unlikely to be the principal factor supporting high abundance of *Endozoicomonas* across numerous coral species (Chase and Hoogenboom 2019). Potentially, these shifts are instead related to the different nutrient balances observed (i.e., carbon:nitrogen:phosphorous) when fish are present in the tank instead of simply supplying their waste water, as highlighted in Neil et al. (2025).

Unlike direct fish associations, there is evidence that dissolved nutrient balances and heterotrophic feeding can influence a coral's microbiome. Galand et al. (2020) found that the deep‐water coral *Madrepora oculata* was able to maintain high *Endozoicomonas* abundance in aquaria when they were supplied with a diatom diet and hypothesised that this was due to the supplied heterotrophic nutrition being most similar to the coral's natural diet. Nutrient enrichment, particularly nitrogen, can alter a coral's microbiome, though past studies have found that *Endozoicomonas* can persist through short‐term changes in dissolved nutrient availability (Deignan and McDougald 2022; Pogoreutz et al. 2018; Rice et al. 2019). Neil et al. (2025) showed that there were differences in the dissolved nutrient profile of tanks that were supplied water from a fish tank, tanks with fish within them, and tanks with fish and a supply of live feeds. In particular, the C:N:P ratio in the LiveFeeds + Fish tanks (calculated as 484:10:1, see Table S2) was closest to the Redfield ratio (106:16:1, (Redfield 1958)) out of any of the treatments and also maintained the highest abundance of *Endozoicomonas*. Potentially, the combination of the live feeds and the fish‐derived nutrients contributed to forming a nutrient balance appropriate to sustain *Endozoicomonas* populations, as it has been suggested that the maintenance of the coral‐*Endozoicomonas* association may require specific nutrient conditions (Pogoreutz and Ziegler 2024). Further work is required to identify what factors influence the stability of the corals' microbiome and promote the high abundance of *Endozoicomonas* taxa across many coral species. Nevertheless, the maintenance of a more field‐like microbial community in tanks with fish highlights a previously unknown potential benefit from co‐culture of corals and fish.

Protein concentration of the corals was highest in the LiveFeeds treatment, followed by the treatments with access to fish waste (Fish, Dissolved and LiveFeeds + Fish). These results closely reflect the previously reported patterns of growth (Neil et al. 2025), which found that access to live feeds and/or fish wastes resulted in higher *P. verrucosa* growth than unfed corals. This is likely due to the increased availability of heterotrophic nutrients, particularly protein, supplied by the live feeds (Conlan, Bay, et al. 2018; Ferrier‐Pagès et al. 2003; Houlbreque and Ferrier‐Pages 2009; Huang et al. 2020) and enrichment of corals' symbionts from the nitrogen and phosphorus in the dissolved fish wastes, increasing photosynthate passage to the host corals (Ezzat et al. 2015; Grover 2002; Muller‐Parker et al. 1994; Wiedenmann et al. 2023). These results are consistent with other studies showing high protein is characteristic of actively growing regions within *Acropora millepora* (Conlan, Humphrey, et al. 2018). Such improvements may also be synergistic with the provision and composition of dietary lipids, as previous research has found that access to an artificial PUFA diet rich in animal protein could increase *Goniopora columna* protein content and specific growth (Aragão et al. 2004; Ding et al. 2021). This is supported by the present study, where LiveFeeds corals, with access to PUFA and protein‐rich *Artemia* nauplii, had the highest protein content. However, increased availability of inorganic nitrogen has also been reported to lead to changes in protein content of Symbiodiniaceae within the tissues of corals (Oakley et al. 2023), which may have contributed to the increase in coral holobiont protein density in some treatments.

Unfed Control nubbins had the lowest total lipid content, in line with previous findings that at lower light levels (as in this experiment), calcification may be sustained whilst tissue mass and lipid content decrease (Anthony and Fabricius 2000). The presence of live feeds (LiveFeeds and LiveFeeds + Fish) resulted in corals with lower total lipid levels than corals without live feeds (Dissolved, Fish or Pellets treatments). Higher growth of *P. verrucosa* in the LiveFeeds treatment relative to other treatments (Neil et al. 2025) suggests that dietary energy from heterotrophic feeding are primarily directed to growth rather than retained in coral tissue as lipid (Ferrier‐Pagès et al. 2003; Treignier et al. 2008). Furthermore, enrichment of coral symbionts via inorganic nitrogen (i.e., ammonium) provision has been linked with increasing translocation of carbon‐rich photosynthates to the coral, which may be stored in tissues as lipids (Dellisanti et al. 2023; Ezzat et al. 2015; Patton and Burris 1983). This association with ammonium provision and lipid storage may partly explain the increased total lipid content observed in coral tissue from the Fish and Dissolved treatments. However, it should be noted that changes in lipid content and composition within the symbionts themselves may have also contributed to the observed differences (Zhang et al. 2023).

Total lipid concentration and composition in corals are known to vary seasonally, with both total lipid and storage lipid content typically increasing in the lead up to spawning events, and subsequently declining after spawning (Cirino et al. 2021; Conlan et al. 2020; Oku et al. 2003; Stimson 1987). Although total lipid content within Field samples was similar to some of the captive treatments, all Field corals had significantly higher storage lipid content, particularly TAG; an important storage lipid that can make up to 37% of lipid content in *P. verrucosa* . TAG is readily catabolised for energy, with resulting low levels indicative of stress (Benson et al. 1978; Harland et al. 1993; Rodrigues et al. 2008). Fragmentation is physiologically challenging for corals, thus low levels of TAG in Post‐acclimation corals may be due to TAG catabolism to fuel recovery and regrowth after fragmentation, paired with an insufficient supply to replenish these stores from their symbionts' activity or heterotrophic feeding (Dornelas et al. 2017; Lirman 2000; Madin et al. 2020).

Despite sustained growth of coral nubbins, after 3 months of being subjected to the diet treatments, total TAG concentration remained relatively low in all corals (~3–6 mg g^−1^ AFDW), with a concomitant increase in the proportion of phospholipid in most treatments. The only treatment for which corals were found to significantly increase the TAG proportion of their total lipids was those from the Control treatment. Harland et al. (1992) found that starved anemones would increase their proportion of storage lipid; however, it was suggested that this was due to a decline in structural lipids, including phospholipids, due to cellular catabolism. Although the TAG *proportion* within Control corals increased (~6% of lipid content), the total *concentration* of TAG relative to AFDW remained similar to other treatments (~6 mg g^−1^ AFDW), giving credence to the idea that this proportional increase was merely a result of a decline in phospholipids.

Heterotrophic feeding can supply corals with the energy and nutrients required to synthesise lipids; however, corals have a relatively low dietary energy requirement compared to asymbiotic animals due to high supplementation from their algal symbionts (Muscatine 1990). Coral's utilisation of heterotrophy is highly contextual, depending on factors such as stress, environmental conditions and individual colony behaviour (Anthony and Fabricius 2000; Grottoli et al. 2006; Teece et al. 2011). In this experiment, the lipid class profile of corals that were supplied live feeds suggests that they preferentially incorporated available phospholipids and/or their constituents rather than TAG. This may be due to sub‐optimal environmental conditions causing corals to direct energy towards growth or to maintain membrane function during physiologically stressful culture periods rather than the proliferation of energy stores (Conlan et al. 2019; Harland et al. 1992; Teece et al. 2011).

Fatty acid profiles of corals reflected the diets available to them, as coral supplied with live feeds (LiveFeeds, LiveFeeds + Fish and Post‐acclimation) had a significantly higher concentration of 18:3n‐3 and 18:1n‐7, fatty acids found in high concentration in *Artemia* (see Figure S1). Although this provides some more direct evidence that the corals were actively feeding on the *Artemia*, analysis of this data showed no evidence that the corals were similarly feeding on the rotifers or supplied phytoplankton. Potentially this was due to the smaller size of these plankton (~140 μm and ~2–25 μm respectively) compared to the 200–400 μm size class of plankton that *Pocillopora* will readily feed upon (Palardy et al. 2005, 2006). Despite *Artemia* being larger (~650 μm) than this preferred size class, past research has demonstrated *Pocillopora* will readily catch and consume them in captivity (Conlan, Bay, et al. 2018; Kuanui et al. 2016; Toh et al. 2013).

The separation between Field corals and captive fragments fatty acid composition was driven primarily by 20:1n‐9, 18:1n‐9, 16:0, 20:3n‐6, 20:0 and 18:1n‐9t. Both 18:1n‐9 and 20:1n‐9 have previously been identified as indicative of zooplankton consumption (e.g., copepods), highlighting the effect different food sources can have on coral fatty acid composition (Radice et al. 2019). However, palmitic acid (16:0) was the major driver of this separation and is the major saturated fatty acid constituent of TAG sourced from coral symbionts (Figueiredo et al. 2012; Kim et al. 2021; Latyshev et al. 1991; Papina et al. 2003; Zhukova 2007). The higher levels of 16:0 in field samples compared to captive corals support the hypothesis that lower light levels in our experiment (and corresponding changes in symbiont activity) contributed to the lack of recovery of TAG stores after fragmentation, resulting in lower TAG concentrations compared to field counterparts (Harland et al. 1993; Yamashiro et al. 1999). Furthermore, PUFA were in general present in higher concentrations in captive corals compared to field corals, and it has previously been suggested that higher PUFA may be associated with shifts in Symbiodiniaceae metabolism under lower light conditions (Rocker et al. 2019; Zhukova 2007).

Overall *P. verrucosa* lipid and fatty acid composition appears primarily influenced by the activity of their symbionts, though heterotrophic sources still played an important role. Symbionts have previously been identified as a primary source for many of the lipids incorporated and metabolised by corals, but heterotrophic feeding can be a significant source of essential omega‐3 fatty acids and energy sources (Teece et al. 2011). It has been suggested that a higher relative n‐3 LC PUFA to n‐6 LC PUFA ratio can be an indicator of increased coral health and may be linked to increased growth and stress resilience (Bachok et al. 2006; Rocker et al. 2019). Rocker et al. (2019) suggested that one way corals maintain these ratios at sites with lower water quality could be through higher relative contributions of heterotrophic feeding. In general, the Field corals in the present study had lower n3:n6 ratios than captive corals, whilst LiveFeeds corals had higher levels of n‐3 LC PUFA relative to the other treatments. Thus, live feeds could be a potential avenue to improve coral growth and stress resilience in captivity via supplementation of n‐3 LC PUFA. It should be noted, however, that factors such as seasonal variation, Symbiodiniaceae density and clade diversity can also influence the relative levels of these fatty acids; thus, the authors cannot preclude that the observed shifts in fatty acid composition in corals in the present study may primarily be a function of the higher symbiont densities sustained in the captive corals (Conlan et al. 2020; Kim et al. 2021).

## 5 Conclusions

This study demonstrates that co‐culturing *Pocillopora verrucosa* with fish, particularly when supplemented with live feeds, can help maintain a physiological and microbial state more closely resembling corals from their natural environment. These findings highlight a promising, low‐effort strategy to enhance coral holobiont condition in aquaculture, with implications for restoration and commercial supply. Future research should investigate species‐specific responses, the application of different husbandry conditions, live feeds varieties and formulated feed compositions, as well as explore the digestive capacity and feed preferences of a variety of cultured coral species.

## Author Contributions

**Rachel C. Neil:** conceptualization, investigation, writing – original draft, methodology, visualization, writing – review and editing, formal analysis, data curation. **Jonathan A. Barton:** investigation, writing – review and editing. **Andrew Heyward:** conceptualization, funding acquisition, methodology, resources, supervision, writing – review and editing. **David S. Francis:** methodology, writing – review and editing. **Leo Nankervis:** methodology, writing – review and editing. **Thomas S. Mock:** methodology, writing – review and editing. **Craig Humphrey:** conceptualization, funding acquisition, methodology, resources, supervision, writing – review and editing. **David G. Bourne:** conceptualization, funding acquisition, methodology, resources, supervision, writing – review and editing.

## Funding

This project was part of The Reef Restoration and Adaptation Program, funded by the partnership between the Australian Governments Reef Trust and the Great Barrier Reef Foundation.

## Ethics Statement

All research was conducted in accordance with the Great Barrier Reef Marine Park Authority permit (G12/35236.1) and James Cook University Animal Ethics Permit (A2787).

## Conflicts of Interest

The authors declare no conflicts of interest.

## Supporting information


**Table S1:** Bayesian model parameters.
**Table S2:** Water quality C:N:P ratios calculated from data in Neil et al. (2025).
**Figure S1:** Principal Components Analysis of (a) fatty acid composition and (b) lipid class data for the different diets fed to the corals, showing ellipses of 95% confidence intervals and the 20 most influential fatty acids as arrows.

## Data Availability

Data and code are available via https://github.com/blue‐bio/pocillopora_fish_nutrition_public. Data is also available via the AIMS Data Centre https://doi.org/10.25845/V8MN‐TQ79. The 16S amplicon sequencing data are available in the NCBI Sequence Read Archive (SRA; https://www.ncbi.nlm.nih.gov/sra) under the BioProject accession number PRJNA1155229.
