# Supplementary material for: Nutritional and Microbial Responses of Pocillopora verrucosa to Co‐Culture With Chromis viridis Damselfish
Source: Environ Microbiol Rep. 2026 Feb 6;18(1):e70291. doi: 10.1111/1758-2229.70291 (PMC12881704; doi:10.1111/1758-2229.70291)
Supplement: Supplementary file 1 — Table S1: Bayesian model parameters. Table S2: Water quality C:N:P ratios calculated from data in Neil et al. (2025). Figure S1: Principal Components Analysis of (a) fatty acid composition and (b) lipid class data for the different diets fed to the corals, showing ellipses of 95% confidence intervals and the 20 most influential fatty acids as arrows. [file EMI4-18-e70291-s001.docx]

**Supplementary Information: Nutritional and microbial responses of *Pocillopora verrucosa* to co-culture with *Chromis viridis* damselfish**

| **Supplementary Table 1: Bayesian model parameters** | | | | | | |
| --- | --- | --- | --- | --- | --- | --- |
| **Response variable** | **Model form** | **Distribution** | **Link** | **Priors** | **Iterations (warmup)** | **Thinning** |
| **Ash** | ash_DW_mgg ~ treatment + (1\|tank_rep_rand), sigma ~ genotype | Gaussian | identity | prior(normal(936, 18), class = 'Intercept') +  prior(normal(0, 20), class = 'b') +  prior(student_t(3, 0, 16), class = 'b', dpar = 'sigma') +  prior(student_t(3, 0, 16), class = 'sd') | 7000  (2500) | 10 |
| **Protein** | protein_AFDW_mgg ~ treatment + (1\|tank_rep_rand:genotype) | Gaussian | log | prior(normal(4, 0.5), class = 'Intercept') +  prior(normal(0, 1), class = 'b') +  prior(student_t(3, 0, 0.5), 'sigma')+  prior(student_t(3, 0, 0.5), class = 'sd') | 7000  (2500) | 7 |
| **Total Lipid** | lipid_AFDW_mgg ~ treatment + (1\|tank_rep_rand:genotype) | Gamma | log | prior(normal(4.5, 0.3), class = 'Intercept') +  prior(normal(0, 1.5), class = 'b') +  prior(student_t(3, 0, 0.3), class = 'sd') +  prior(gamma(2, 1), class = 'shape') | 7500  (2500) | 10 |
| **SE** | SE ~ treatment + (1\|tank_rep_rand:genotype) | Gaussian | identity | prior(normal(10.9, 3), class = 'Intercept') +  prior(normal(0, 6), class = 'b') +  prior(student_t(3, 0, 3), class = 'sd') +  prior(student_t(3, 0, 3), class = 'sigma') | 5000  (2500) | 10 |
| **TAG** | TAG ~ treatment + (1\|tank_rep_rand:genotype) | Gamma | log | prior(normal(1.8, 0.4), class = 'Intercept') +  prior(normal(0, 1.5), class = 'b') +  prior(student_t(3, 0, 0.4), class = 'sd') +  prior(gamma(0.01, 0.01), class = 'shape') | 5000  (2500) | 10 |
| **1,2-DAG** | DAG ~ treatment + (1\|tank_rep_rand:genotype), sigma ~ treatment | Gaussian | identity | prior(normal(8.9, 3), class = 'Intercept') +  prior(normal(0, 5), class = 'b') +  prior(student_t(3, 0, 1.1), class = 'sd') +  prior(student_t(3, 0, 1.1), class = 'b', dpar = 'sigma') | 10000  (2500) | 15 |
| **FFA** | FFA ~ treatment + (1\|tank_rep_rand:genotype) | Gaussian | identity | prior(normal(3.5, 1.5), class = 'Intercept') +  prior(normal(0, 4), class = 'b') +  prior(student_t(3, 0, 1.2), class = 'sd') +  prior(student_t(3, 0, 1.2), class = 'sigma') | 7000  (2500) | 5 |
| **STEROL** | STEROL ~ treatment + (1\|tank_rep_rand:genotype) | Gaussian | identity | prior(normal(6.0, 2), class = 'Intercept') +  prior(normal(0, 3.5), class = 'b') +  prior(student_t(3, 0, 1), class = 'sd') +  prior(student_t(3, 0, 1), class = 'sigma') | 10000  (2500) | 10 |
| **AMPL** | AMPL ~ treatment + (1\|tank_rep_rand), shape ~ treatment*genotype | Gamma | log | prior(normal(3.1, 0.5), class = 'Intercept') +  prior(normal(0, 1), class = 'b') +  prior(student_t(3, 0, 0.3), class = 'sd') +  prior(normal(0, 1), class = 'b', dpar = 'shape') | 5000  (2500) | 5 |
| **PE** | PE ~ treatment + (1\|tank_rep_rand:genotype), sigma ~ treatment | Gaussian | identity | prior(normal(8.8, 3), class = 'Intercept') +  prior(normal(0, 4), class = 'b') +  prior(student_t(3, 0, 1), class = 'sd') +  prior(normal(0, 1), class = 'b', dpar = 'sigma') | 7000  (2500) | 5 |
| **PSPI** | PSPI ~ treatment + (1\|tank_rep_rand:genotype), sigma ~ treatment | Gaussian | identity | prior(normal(13.0, 4), class = 'Intercept') +  prior(normal(0, 9), class = 'b') +  prior(student_t(3, 0, 1.5), class = 'sd') +  prior(normal(0, 1.5), class = 'b', dpar = 'sigma') | 8000  (2500) | 10 |
| **PC** | PC ~ treatment + (1\|tank_rep_rand:genotype), sigma ~ treatment | Gaussian | identity | prior(normal(11.3, 4), class = 'Intercept') +  prior(normal(0, 6), class = 'b') +  prior(student_t(3, 0, 1.5), class = 'sd') +  prior(normal(0, 1.5), class = 'b', dpar = 'sigma') | 8000  (2500) | 10 |
| **LPC** | LPC ~ treatment + (1\|tank_rep_rand:genotype), sigma ~ treatment | Gaussian | identity | prior(normal(6.7, 3), class = 'Intercept') +  prior(normal(0, 4), class = 'b') +  prior(student_t(3, 0, 1), class = 'sd') +  prior(normal(0, 1), class = 'b', dpar = 'sigma') | 8000  (2500) | 10 |
| **Storage** | Storage ~ treatment + (1\|tank_rep_rand:genotype), sigma ~ treatment | Gaussian | identity | prior(normal(30, 6), class = 'Intercept') +  prior(normal(0, 20), class = 'b') +  prior(student_t(3, 0, 6), class = 'sd') +  prior(normal(0, 6), class = 'b', dpar = 'sigma') | 9000  (2500) | 10 |
| **Structural** | Structural ~ treatment + (1\|tank_rep_rand:genotype), sigma ~ treatment | Gaussian | identity | prior(normal(70, 6), class = 'Intercept') +  prior(normal(0, 20), class = 'b') +  prior(student_t(3, 0, 6), class = 'sd') +  prior(normal(0, 6), class = 'b', dpar = 'sigma') | 9000  (2500) | 10 |
| **TAG as proportion of total lipid** | TL_TAG ~ treatment + (1\|tank_rep_rand:genotype | Gamma | log | prior(normal(1.6, 0.9), class = 'Intercept') +  prior(normal(0, 1.3), class = 'b') +  prior(student_t(3, 0, 0.5), class = 'sd') +  prior(gamma(0.01, 0.01), class = 'shape') | 7500  (2500) | 10 |
| **PUFA** | PUFA ~ treatment + (1\|tank_rand:genotype) | Gaussian | identity | prior(normal(32.7, 5), class = 'Intercept') +  prior(normal(0, 20), class = 'b') +  prior(student_t(3, 0, 4.5), class = 'sd') +  prior(student_t(3, 0, 4.5), class = 'sigma') | 7500  (2500) | 10 |
| **MUFA** | MUFA ~ treatment + (1\|tank_rand:genotype),  shape ~ treatment | Gamma | log | prior(normal(1.1, 0.2), class = 'Intercept') +  prior(normal(0, 1), class = 'b') +  prior(student_t(3, 0, 0.2), class = 'sd') +  prior(normal(0, 1), class = 'b', dpar = 'shape') | 7500  (2500) | 10 |
| **SFA** | SFA ~ treatment + (1\|tank_rand:genotype | Gaussian | identity | prior(normal(55.3, 6), class = 'Intercept') +  prior(normal(0, 20), class = 'b') +  prior(student_t(3, 0, 6), class = 'sd') +  prior(student_t(3, 0, 6), class = 'sigma') | 7500  (2500) | 10 |
| **PUFA n-3:n-6** | PUFA_n3_n6 ~ treatment + (1\|tank_rand:genotype), shape ~ treatment | Gamma | log | prior(normal(0.6, 1), class = 'Intercept') +  prior(normal(0, 0.5), class = 'b') +  prior(student_t(3, 0, 1), class = 'sd') +  prior(normal(0, 0.6), class = 'b', dpar = 'shape') | 7500  (2500) | 10 |
| **LC PUFA n-3:n-6** | LC_n3n6 ~ treatment + (1\|tank_rand:genotype), shape ~ treatment | Gamma | log | prior(normal(0.7, 1), class = 'Intercept') +  prior(normal(0, 0.5), class = 'b') +  prior(student_t(3, 0, 1), class = 'sd') +  prior(normal(0, 0.5), class = 'b', dpar = 'shape') | 7500  (2500) | 10 |


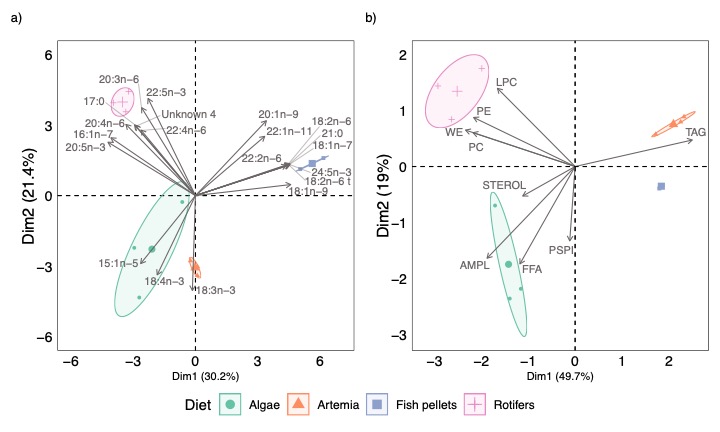


Supplementary Figure 1: Principal Components Analysis of a) fatty acid composition and b) lipid class data for the different diets fed to the corals, showing ellipses of 95% confidence intervals and the twenty most influential fatty acids as arrows.

LCP = Lysophosphatidylchloline, PC = Phosphatidylcholine, PSPI = Phosphatidylserine and Phosphatidylinositol, PE = Phosphatidylethanolamine, AMPL = Acetone mobile polar lipids, Sterol = Sterol, FFA = Free fatty acids, TAG = Triacylglyceride, WE = Wax esters

The supplied diets, including *Artemia,* rotifers, microalgae and fish pellets, differ in their fatty acid and lipid class composition. *Artemia* FA composition was characterised by higher 18:1n-9t, 18:3n-3, 18:1n-7 and 16:3n-4 concentrations (as % of total fatty acids) than the other diets (Supplementary Figure 1a). *Artemia* and the fish pellets also had a much higher proportion of TAG compared to the rotifers and mixed algae (Supplementary Figure 1b). Higher relative concentrations of AMPL characterised the mixed microalgae samples, and both microalgae and rotifers had higher proportions of phospholipids than the *Artemia* and fish pellets.

| **Supplementary Table 2: Water quality C:N:P ratios calculated from data in Neil et al. (2025)** | | | | | | | |
| --- | --- | --- | --- | --- | --- | --- | --- |
| **Treatment** | **DOC** | | **N (µmol/L)** | **PO_4_ (µmol/L)** | **Ratio** | | |
|  | mg/L | µmol (mg/L * 1000 / 12.011) | (NH_4_ + NO_2_ + NO_3_) |  | C | N | P |
| **Control** | 1.13 | 94.08 | 1.079 | 0.169 | 556.7 | 6.4 | 1.0 |
| **Pellets** | 1.11 | 92.42 | 1.383 | 0.171 | 540.4 | 8.1 | 1.0 |
| **LiveFeeds** | 1.13 | 94.08 | 1.212 | 0.178 | 528.5 | 6.8 | 1.0 |
| **LiveFeeds + Fish** | 1.07 | 89.09 | 1.928 | 0.184 | 484.2 | 10.5 | 1.0 |
| **Fish** | 1.13 | 94.08 | 1.626 | 0.179 | 525.6 | 9.1 | 1.0 |
| **Dissolved**  **Coral tank** | 1.16 | 96.58 | 1.128 | 0.141 | 685.0 | 8.0 | 1.0 |
| **Dissolved**  **Fish tank** | 1.22 | 101.57 | 1.665 | 0.175 | 580.4 | 9.5 | 1.0 |

Neil, R. C., Barton, J. A., Heyward, A., Francis, D. S., Nankervis, L., Mock, T. S., Bourne, D. G., & Humphrey, C. (2025). Improving Coral Grow-Out Through an Integrated Aquaculture Approach. *Aquaculture Nutrition*, *1446195*, 13 pages. <https://doi.org/https://doi.org/10.1155/anu/1446195>
